# Quality of life and societal costs in hypertrophic cardiomyopathy: protocol of the AFFECT-HCM study

**DOI:** 10.1007/s12471-022-01753-0

**Published:** 2023-01-16

**Authors:** Stephan A. C. Schoonvelde, Isabell Wiethoff, Mickaël Hiligsmann, Silvia M. A. A. Evers, Michelle Michels

**Affiliations:** 1 Department of Cardiology, Erasmus Medical Centre, University Medical Centre Rotterdam, Rotterdam, The Netherlands; 2 Department of Health Services Research, Care and Public Health Research Institute (CAPHRI), Maastricht University, Maastricht, The Netherlands; 3 Trimbos Institute, Netherlands Institute of Mental Health and Addiction, Centre for Economic Evaluation and Machine Learning, Utrecht, The Netherlands

**Keywords:** Hypertrophic cardiomyopathy, Burden of disease, Healthcare resource use, Cost-of-illness, Quality of life

## Abstract

**Background:**

Ever since the first description of hypertrophic cardiomyopathy (HCM), the most common genetic cardiac disease, tremendous progress has been made in the evaluation and management of HCM patients, but little attention has been focused on the impact of HCM on societal costs and quality of life (QoL).

**Aims:**

This paper describes the study protocol for the AFFECT-HCM study into burden of disease (BoD), which aims to estimate health-related QoL and societal costs in HCM patients and genotype-positive phenotype-negative (G+/P−) relatives during a one-year follow-up study, and relate this to the phenotypical HCM expression.

**Methods:**

A total of 400 Dutch HCM patients and 100 G+/P− subjects will be followed for one year in a prospective, multi-centre, prevalence-based BoD study. Societal costs will be measured via a bottom-up approach using the cost questionnaires iMCQ and iPCQ. For QoL, the generic EQ-5D-5L and disease-specific Kansas City Cardiomyopathy Questionnaire will be used. QoL and societal costs will be compared with phenotype-specific HCM characteristics and other determinants to identify factors that influence BoD. Accelerometry will test the correlation between BoD and physical activity.

**Conclusion:**

The AFFECT-HCM study will evaluate the BoD in HCM patients and G+/P− subjects to improve the understanding of the societal and economic impact of HCM.

## What’s new?

- The AFFECT-HCM study will be the first Dutch study looking to quantify QoL and societal costs in HCM patients and G+/P− subjects.
- It will provide novel insight into the influence of HCM on QoL perception and costs associated with HCM.
- Physical activity will be tracked and possible correlations of physical activity in combination with HCM on QoL perception and costs will be investigated.
- Important knowledge on the societal and economic impact of HCM will be acquired, which may assist in guiding future healthcare system and policy decisions, and help design economic evaluations.

## Introduction

Hypertrophic cardiomyopathy (HCM) is the most common autosomal dominant genetic cardiac disease, affecting an estimated 1 in 200 to 500 people worldwide [1, 2]. It is characterised by left ventricular hypertrophy in the absence of abnormal loading conditions. It may cause symptoms and events such as dyspnoea, angina, palpitations, syncope or sudden cardiac death [3]. In the majority of cases, HCM is a monogenic disease and therefore family screening is recommended. Upon the clinical and, if applicable, genetic diagnosis of HCM, family members are advised to undergo pre-symptomatic genetic or cardiac screening [4]. This leads to the identification of family members carrying the familial pathogenic DNA variant without HCM (genotype-positive, phenotype-negative: G+/P−). These G+/P− subjects undergo regular cardiac evaluation in order to monitor possible HCM development. Thus, in addition to decreased physical, social and psychological functioning that HCM patients face, both HCM patients and G+/P− subjects may also face losses in quality of life (QoL) through external and internal stressors and anxiety [5, 6]. Consequently, HCM creates a social and economic burden for affected individuals and their relatives.

Burden of disease (BoD) studies measure the economic and societal impact of health conditions and gather valuable information for healthcare policy and decision-making [7]. Disease burden is often quantified in QoL expressed in utilities, as well as in societal costs captured in monetary units [8]. In addition to classical epidemiological measures and mortality, QoL and societal costs are increasingly recognised as central denominators of the disease burden [9]. However, within the current literature, not much research has been done investigating the QoL and societal costs in patients with HCM and G+/P− family members. Recent cost-of-illness studies have only estimated healthcare costs, leaving out information about broader cost types [10–12]. Furthermore, QoL studies are rare and none have measured QoL with the EQ-5D-5L, the required instrument according to Dutch guidelines [5, 13–15].

The AFFECT-HCM (Quality of Life and Costs in HCM) study aims to estimate the one-year impact of HCM on generic and disease-specific QoL and the societal costs of HCM in the Netherlands. The study will further explore the relationship between costs/QoL and phenotypic as well as socio-demographic characteristics. Moreover, physical activity in HCM patients and G+/P− subjects will be evaluated and linked to phenotype, QoL and cost outcomes. To our current knowledge, this will be the first BoD study performed in a Dutch setting that assesses broader cost types of HCM and the first BoD study that in addition to HCM patients also includes G+/P− relatives.

## Methodology

### Study design

**Fig. 1:**
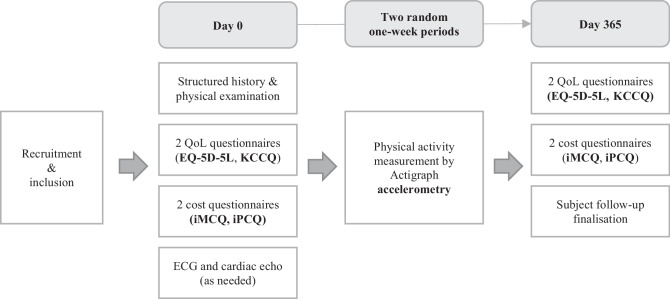
The structured timeline of the study per subject. *QoL* quality of life, *EQ-5D-5L* EuroQol questionnaire, *KCCQ* Kansas City cardiomyopathy questionnaire, *iMCQ* medical consumption questionnaire, *iPCQ* productivity cost questionnaire

The AFFECT-HCM is a multi-centre prospective observational cohort study assessing the BoD of HCM patients and G+/P− relatives. Costs and QoL will be measured following a prevalence-based approach including subjects with various phenotypic expressions at different disease stages. Data will be collected at baseline and during follow-up after one year. Patients will be recruited from their respective hospitals for baseline analyses. The subject inclusion session will contain four questionnaires and clinical analyses consisting of a structured history and a physical examination. An electrocardiogram and echocardiography will be performed if these have not been performed within the last six months before the patient inclusion date. A physical activity tracker (ActiGraph), also known as an accelerometer, will be provided during two separate one-week intervals to monitor physical activity, assessing its possible influence and relationship on QoL perception and disease costs. A structured timeline is provided (Fig. 1).

### Study population

**Fig. 2:**
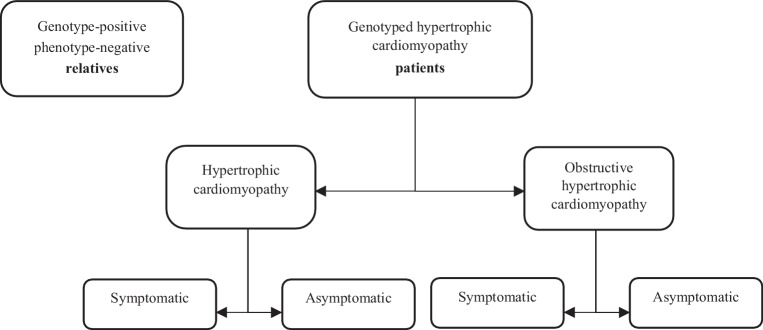
Group and subgroup division of included subjects. Groups are divided into two main groups: genotype-positive phenotype-negative family relatives and hypertrophic cardiomyopathy (HCM) patients who have had prior genotyping. Patients are in turn divided into non-obstructive HCM and obstructive HCM. Both subgroups are further subdivided into symptomatic and asymptomatic patients

Subjects will be divided into two main groups: phenotype-positive HCM patients and G+/P− relatives (Fig. 2). HCM patients must have undergone prior genetic testing and may be either G+ or genotype-negative (G−). HCM patients will be further subdivided into either non-obstructive or obstructive HCM (left ventricular outflow tract gradient of ≥30 mm Hg). Both of these subgroups will in turn be split into symptomatic (New York Health Association [NYHA] class II to IV) and asymptomatic patients (NYHA I).

### Recruitment

Genotyped HCM patients and G+/P− subjects aged 18–80 year will be recruited consecutively at dedicated cardio-genetic outpatient clinics in participating university hospitals that are part of the Double Dose research consortium and from cardiomyopathy databases in participating centres. Additionally, the Dutch patient advocacy group for HCM will distribute study information through their communication channels, allowing for patients from other hospitals to be included.

#### Inclusion criteria

- Genotyped HCM patients
- Or G+/P− family members
- Ages 18–80 years old
- Proficiency with the Dutch language

#### Exclusion criteria

- Any subject not able to provide informed consent or fill in the questionnaires

### Sample size

Due to the absence of sample size calculation methods for BoD studies, the sample size was based on previously conducted studies with similar designs indicating that a number of approximately 200 participants is sufficient [16–18]. Given the high prevalence of HCM, the low study burden for subjects, the number of participating academic HCM centres and the interest of carrying out subgroup analyses, a total number of 500 participants (consisting of approximately 400 HCM patients and 100 G+/P− carriers) is targeted and deemed feasible. As an underlying genetic variant can be found in 30–50% of the clinically diagnosed cases, the sample size of the G+ HCM group may be correspondingly smaller [19].

### Data collection

#### Cost assessment

Costs will be estimated from a societal perspective including medical costs, patient and family costs, productivity losses, and costs outside the healthcare sector (Tab. 1). Therefore, corresponding healthcare resource utilisation of patients and relatives will be captured at an individual level via a bottom-up approach by using the iMCQ (medical consumption questionnaire) and the iPCQ (productivity cost questionnaire). The iMCQ entails 20 questions about the healthcare resource utilisations of patients for a broad range of healthcare services, while the iPCQ contains 12 questions about the lost productivity for paid and unpaid work. The monetary valuation of the used healthcare will be done with the Dutch costing tool by assigning reference prices to each resource [15]. For long-term productivity losses, the friction cost method will be used as recommended in the Dutch Manual for Costing [15]. As the study period does not exceed one year, discounting is not required.

**Table 1:** Costs included in AFFECT-HCM study shown by cost types

Medical costs	Patient & family costs	Productivity losses	Costs in other sectors
– General practitioner – Social worker – Practice assistant – Physiotherapy – Occupational therapy – Speech therapy – Dietician – Homeopathy – Psychologist – Company physician – Medications – Examinations – Specialist care – Hospitalisations – Overnight stays and care received at other facilities	– Home care (household, self-care, nursing) done by family member – Traveling time to care facilities	– Lost working hours for paid work (absenteeism) – Reduced productivity at work (presenteeism)	– Lost working hours for unpaid/voluntary work

#### Quality of life assessment

Generic QoL of patients and G+/P− relatives will be measured with the Dutch version of the standardised and validated EQ-5D-5L questionnaire as preferred by the Dutch guideline [15]. The EQ-5D-5L captures five health dimensions (mobility, self-care, usual activities, pain/discomfort, and anxiety/depression), each with five answer levels resulting in 3125 possible health profiles [20]. These health profiles will be valued with the Dutch EQ-5D-5L value set in order to derive utility scores. Utilities are comparable between diseases and may be expressed in a score between zero (death) and one (perfect health). After a year, quality-adjusted life years (QALYs) can be derived as this measure combines the quality of life (utility) with the length of life [15, 21].

Disease-specific QoL will be measured with the Kansas City Cardiomyopathy Questionnaire (KCCQ), which covers seven health domains (symptom frequency, symptom burden, symptom stability, physical limitations, social limitations, quality of life, and patient self-efficacy) [22]. All dimensions range from zero (worst functioning) to 100 (excellent health). The KCCQ has been proven reliable and sensitive to monitor clinical status, and has been validated for HCM [13, 23, 24].

#### Physical activity measurement

The ActiGraph accelerometer (Model wGT3X-BT; ActiGraph, LLC., Pensacola, Florida, USA) will be used to objectively measure the volume of physical activity of each subject. An ActiGraph accelerometer is designed to detect body movements. It has been validated for medical research in adults [25, 26]. The accelerometer will be worn for two separate one-week periods (worn on a belt around the hip), except when bathing.

### Outcomes

#### Primary outcomes

- Societal costs of HCM and G+/P− status will be adjusted for inflation and expressed in euro of the respective reporting year. Baseline costs will be extrapolated to one year and described as costs per patient per year. After study completion, longitudinal cost data will be analysed in order to observe potential cost developments or trends.
- Generic QoL will be reported in utilities and disease-specific QoL will be shown on a 0–100 scale. QALYs will be calculated and reported.

#### Secondary outcomes

- The potential relationship between costs, QoL and phenotypic as well as socio-demographic characteristics will be analysed with correlation and regression techniques.
- Potential differences in costs and QoL between phenotype status (HCM vs G+/P− subjects) and disease subtypes (non-obstructive HCM vs obstructive HCM), and therein symptomatic versus asymptomatic patients, will be explored with subgroup analyses (Tab. 2).
- Physical activity between the subgroups will be evaluated by accelerometry, which will be linked to QoL and cost outcomes.

**Table 2:** Subgroup analyses plan

Costs and quality of life/utility analysed by:	
*Overall cohort*	*n* ~ 500
*Patient characteristics*	
– Age	By age groups
– Sex	Men vs women
– Socio-economic status	Level of education, i.e. low, middle, high
*Clinical parameter*	
– Symptomatology	Asymptomatic (NYHA class I) vs symptomatic (NYHA class II to IV)
– Disease subtype	HCM vs oHCM (left ventricular outflow tract gradient of ≥ 30 mm Hg)

*NYHA* New York Heart Association classification, *HCM* hypertrophic cardiomyopathy, *oHCM* obstructive hypertrophic cardiomyopathy

### Statistical analysis

Statistical analysis will be performed with baseline patient characteristics at one year and with longitudinal data after follow-up of the last patient following finalisation of the study. We will use IBM SPSS Statistics version 25 (SPSS, IBM, Armonk, New York). Patient characteristics and baseline outcomes will be analysed with descriptive methods by providing relative frequencies for categorical data, mean and standard deviations for normally distributed continuous data, or median and interquartile ranges in case of non-normality. Normality of data will be tested using the Shapiro–Wilk test. As the cost-distribution is expected to be non-normal, bootstrapping (1000 simulations) will be performed to calculate 95% confidence intervals [27]. Missing data will be considered irrelevant for further analysis and excluded.

Potential correlations between costs and other variables, such as age or disease severity, will be analysed using the non-parametric Kendall’s tau‑b correlation coefficient. Furthermore, we will use multiple regression to identify variables that are associated with QoL. Moreover, subgroup differences in costs and QoL will be analysed with the chi-squared (χ^2^) test for categorical data, the independent samples t‑test for normally distributed continuous data, and the Mann-Whitney U test for non-normally distributed continuous data, and will be reported for each subgroup respectively. The planned subgroup analyses are summarised in Tab. 2. For accelerometry measurements, the activity intensity, i.e. the number of minutes per hour of moderate to vigorous physical activity (> 3 metabolic equivalents), will be calculated using a threshold value of 2100 counts per minute [26]. Activity counts per hour shall be modified to counts per minute to provide a measure of activity volume, which allows for comparison with other studies. Between-group results will be compared using a two-way analysis of variance to test for main effects between the groups. Furthermore, one-way sensitivity analyses will be performed. Base case assumptions for cost prices will be varied to analyse their impact on the results. Lastly, costs will also be calculated from a healthcare perspective.

### Ethics

The study will be conducted according to the principles of the Declaration of Helsinki and the ISO 14155 Good Clinical Practice guidelines for medical devices. The study does not fall under the scope of the Medical Research Involving Human Subjects Act and has been approved by the Institutional Review Board of the Erasmus University Medical Centre Rotterdam (MEC-2022-0036).

## Discussion

The AFFECT-HCM study will be the first Dutch BoD study of HCM. It sets out to measure QoL and societal costs in HCM patients and G+/P− subjects during a one-year follow-up period. Due to the clinical heterogeneity of HCM, the disease burden is assumed to vary between the previously defined subgroups. Furthermore, this study is expected to provide valuable insights into QoL perception and mental wellbeing of G+/P− variant carriers. This is important since carriers may fear developing HCM with its associated health consequences, something which may have been observed in affected family members. The study will also collect novel information about the economic burden of HCM, in addition to evaluating costs beyond healthcare, as these broader costs are considerably less explored.

The AFFECT-HCM study has some limitations. Firstly, the planned bottom-up approach will use patient-reported outcomes, i.e. data based on information given by participants. This might be subjective and may sometimes be based on estimations. However, previous cost-of-illness studies used aggregated databases and thus neglected important information about broader costs such as patient and family costs, productivity losses and costs beyond healthcare, which are included in this study [10–12]. Secondly, the to-be-used questionnaires are validated for an adult population only, hence minors will be excluded [20]. Thirdly, potential recall-bias due to the retrospective measurement of healthcare resources may take place. In order to minimise potential recall-bias, whilst ensuring a high informative value, a recall-period of three months is regarded as optimal [28].

This study protocol will be the first Dutch prospective cohort study in HCM patients and G+/P− carriers to provide insights into different methods for estimating the BoD, which could provide a framework to guide future research. It will also provide important patient-specific information for economic evaluations to analyse different care strategies and to further optimise care for HCM patients. To improve the understanding of the disease impact, future research into the BoD in HCM should be performed in more settings.
